# Different Polymers for the Base of Removable Dentures? Part I: A Narrative Review of Mechanical and Physical Properties

**DOI:** 10.3390/polym15173495

**Published:** 2023-08-22

**Authors:** Pierre Le Bars, Octave Nadile Bandiaky, Laurent Le Guéhennec, Roselyne Clouet, Alain Ayepa Kouadio

**Affiliations:** 1 Department of Prosthetic Dentistry, Faculty of Dentistry, Nantes University, 1 Place Alexis Ricordeau, 44042 Nantes, France; laurent.leguehennec@univ-nantes.fr (L.L.G.); roselyne.clouet@univ-nantes.fr (R.C.); ayepa_alain@yahoo.fr (A.A.K.); 2 Oniris, CHU Nantes, INSERM, Regenerative Medicine and Skeleton, RMeS, Nantes Université, UMR 1229, 44000 Nantes, France; octave.bandiaky@univ-nantes.fr; 3 Department of Prosthetic Dentistry, Faculty of Dentistry, University Hospital Center, Abidjan P.O. Box 612, Côte d’Ivoire

**Keywords:** denture base material, PMMA, polymethylmethacrylate, polyamide, polyetheretherketone, mechanical proprieties

## Abstract

Even before considering their introduction into the mouth, the choice of materials for the optimization of the prosthesis depends on specific parameters such as their biocompatibility, solidity, resistance, and longevity. In the first part of this two-part review, we approach the various mechanical characteristics that affect this choice, which are closely related to the manufacturing process. Among the materials currently available, it is mainly polymers that are suitable for this use in this field. Historically, the most widely used polymer has been polymethyl methacrylate (PMMA), but more recently, polyamides (nylon) and polyether ether ketone (PEEK) have provided interesting advantages. The incorporation of certain molecules into these polymers will lead to modifications aimed at improving the mechanical properties of the prosthetic bases. In the second part of the review, the safety aspects of prostheses in the oral ecosystem (fragility of the undercuts of soft/hard tissues, neutral pH of saliva, and stability of the microbiota) are addressed. The microbial colonization of the prosthesis, in relation to the composition of the material used and its surface conditions (roughness, hydrophilicity), is of primary importance. Whatever the material and manufacturing process chosen, the coating or finishes dependent on the surface condition remain essential (polishing, non-stick coating) for limiting microbial colonization. The objective of this narrative review is to compile an inventory of the mechanical and physical properties as well as the clinical conditions likely to guide the choice between polymers for the base of removable prostheses.

## 1. Introduction

By 2050, the world’s population of older adults is expected to reach 2 billion, which suggests a significant increase in the number of users of removable prostheses [1,2,3]. In view of the great geographical disparity in healthcare, older populations in economically deprived areas are obliged to forego implants as a solution and tend toward rehabilitation by removable prostheses using different polymers. These include polymethyl methacrylate (PMMA; or poly [1-(methoxy carbonyl)-1-methyl ethylene]), which is the most widely used polymer for prosthetic bases [4]. Chemically, PMMA is synthesized through an addition chain reaction coupled with the polymerization of methyl methacrylate. The polymerization reaction can be initiated at room temperature or higher (90–120 °C) by light curing or microwaves [5]. Depending on the polymerization technique, by compression or by injection, the parameters of flexural strength (FS) and flexural modulus (FM) vary. Compression molding has shown its superiority in this area [6] (Figure S1).

In the search for better functional performance, various members of the polymer family have been tested, including polyamide, epoxy, polystyrene, or vinyl-acrylic resins, but without yielding completely satisfactory results [7]. For flexibility and fracture resistance, research showed the significant superiority of polyamide bases over PMMA [8]. The polyamides or “nylons” are thermoplastic polymers resulting from the condensation between a diamine and a dibasic acid. This thermoplastic flexible polyamide resin is composed of long chains with a few cross-linkers between them. The result is a low resistance to surface pressure [9]. In addition, due to its chemical structure, this linear polyamide also has low hardness and reduced abrasion resistance [10].

Another polymer, polyether ether ketone (PEEK), thanks to its ability to resist functional stresses to bending, makes it possible to prolong the clinical use of a denture base while counteracting the more frequent fractures of PMMA bases [11]. PEEK is a semi-crystalline, thermoplastic polymer with a high melting temperature (machined or pressed thermoplastic; 150–300 °C) resulting from the dialkylation of bis phenolates of PEEK monomers [12]. PEEK is mainly shaped by injection molding, by compression, or by extrusion for removable partial dentures (RPD) [13] (Figure S2).

All of these recent advances have improved the mechanical properties of polymer materials and enable new performance concerning the clinical longevity of prostheses. However, there are clear differences between these materials. Therefore, this narrative re-view compiles an inventory of the mechanical, physical properties, and clinical conditions likely to guide the choice between these polymers for the base of a removable partial or complete prosthesis.

## 2. Mechanical Properties of Polymer Biomaterials (PMMA, Polyamide, and PEEK)

The absence of standardization concerning the means and the parameters used to evaluate the mechanical properties of polymers makes it difficult to compare these bio-materials. Comparisons are further complicated by the fact that companies employ different manufacturing methods to produce polymers (PMMA, polyamide, and PEEK), whose characteristics are variable. However, in vitro, if we refer to laboratory investigations only, the risk of bias is minimized, as shown in Table 1.

The success of PMMA is explained by its ease of implementation in the laboratory, by the possibility of straightforward repairs, retouching, or rebasing, and by its low cost. In the mouth, its low saliva absorption, solubility, and toxicity with biological tolerance over the long term, as well as the excellent esthetic outcome, makes it a material of choice. However, PMMA shows polymerization shrinkage, weak FS, low resistance to bending and to shocks, and an inefficient resistance to fatigue [28,29,30,31].

Because of the aforementioned failures, an alternative in the family of polyamides is sought. Among the various commercial polyamides available (Valplast^®^, Corp 200 Shames Drive Westbury, NY, USA; Valplast Flexite ^®^, Flexite company, Mineola, NY, USA; Luci-tone Versacry^®^, Dentsply Sirona, NY, USA; Vertex^®^, Dentimex, Zeist, Netherlands; Bre-flex^®^ and Brecrystal^®^, Bredent medical GmbH & Co. KG, Senden, Germany), the choice is governed by the many advantages of polyamide/PMMA, such as the impact strength (IS). This is partly attributed to the acrylic resin injection technique that is used to mold the prosthetic base. During this phase, polymerization under high pressure by eliminating air bubbles and limiting the reaction of the shrinkage of the resin during setting partly explains the high IS value [32]. On the other hand, the low content of cross-linking agents in the polyamide coupled with a non-negligible amount of residual monomer contributes toward improving the IS [16]. For example, Ucar et al. reported that, in their study, the polyamide (Deflex^®^, Nuxen S.R.L, Buenos Aires, Argentina) material had good FS (MPa = 78 ± 1.0) close to that of PMMA (SR-Ivocap^®^, Ivoclar AG, Schaan, Liechtenstein, Germany; MPa = 69.8 ± 1.4) and Meliodent^®^ (Bayer Co., Leverkusen, Germany; MPa = 81.1 ± 1), but its FM remained lower (GPa = 0.70 ± 0.13) than that of PMMA (SR-Ivocap^®^, GPa = 0.85 ± 0.27) and Meliodent^®^ (GPa = 1.70 ± 0.23) [16]. Concerning Knoop hardness (measured in kg/cm^2^), these authors found values of 7.5 ± 1.0 for Deflex^®^, 13.5 ± 1.4 for SR-Ivocap^®^, and 16.9 ± 1.0 for Meliodent^®^. In these experimental conditions, the Meliodent^®^ specimens fractured during flexural testing, but none of the Deflex^®^ specimens did [16]. Other more recent studies in vitro confirmed this result [5,33,34,35].

Regarding the specificity of the partially removable prosthesis, an alternative to PMMA and metal alloys is possible. In this context, the high-performance PEEK can be chosen as the constituent material for dental prosthesis bases. For a few years, and also more recently, numerous in vitro and in vivo studies have described the noteworthy properties of PEEK with a universal testing machine: The superiority of PEEK/PMMA lies in its FS (183 MPa > 84 MPa) and its hardness, as observed when using the Vickers microhardness tester (24 VHN > 19.4 VHN) over the hardened PMMA at a high polymerizing temperature [11]. In vitro, the modulus of elasticity of PEEK is 3.6 GPa. This can be improved by adding carbon fibers and may reach 18 GPa, which is close to the values of cortical bone (15 GPa) [36,37,38]. However, PEEK has a high risk of fracture and abrasion. More recently, these different characteristics, determined by in vitro studies, mean that the milled or pressed PEEK polymer at a mold temperature of 200 °C has mechanical properties making it suitable for use as a removable partial prosthesis. However, its use in removable prostheses remains limited because of the additional difficulty concerning the best way to bond the PEEK to the artificial teeth; furthermore, progress must also be made concerning the functional aspect of the claps.

Liebermann et al. evaluated, in vitro, the effects of aging on the physical and mechanical properties of several materials (PEEK, PMMA, composite resin, hybrid materials) [39]. Samples were kept in various storage media (distilled water, sodium chloride, saliva, etc.) for 1, 7, 14, 28, 90, and 180 days, after which the roughness, the water absorption, and the solubility had changed. The results showed that PEEK had the lowest solubility and water absorption values, as presented in Table 2 [40]. 

Another study by Takabayashi et al. showed that the water absorption of two polyamides (Valplast and Flexite Supreme) corresponds to the ISO standard (32 μg/mm^3^), while on the other hand, that of Lucitone FRS is higher [21]. This may be related to the hydrophilic characteristics (low contact angle) of this polyamide. Thus, assuming that the concentration of the amide group promotes water absorption, the fact of lowering this level would make it possible to limit the absorption, as is the case with nylon 6 or 66 [39].

To assess the consequences of in vitro aging, concerning dimensional changes and ultimate tensile strength, three polyamide materials were tested for the manufacture of prosthesis bases by injection molding. After a 6-month experimentation period evaluating Biosens^®^ (Perflex, Netanya, Israel), Bre.flex^®^ 2nd edition (Bredent, Senden, Germany), and ThermoSens^®^ (Vertex Dental B.V., Soesterberg, The Netherlands), the ultimate tensile strength was conclusively found for Biosens^®^ and ThermoSens^®^, but with the weakest alterations observed for Bre.flex^®^ [53].

## 3. Different Materials Incorporated in PMMA, Polyamide, and PEEK Improve Their Physical Characteristics

For many defects (low FS, low resistance to shock, and low fracture resistance), some ameliorations of PMMA are still sought today. To this end, the addition of metal wires, plates and fibers and the modification of the chemical structure have been tested, but most experiments have been carried out in vitro and therefore need to be confirmed in vivo. For example, the zirconia (3–5%) or 15% acrylamide monomer incorporated into the PMMA makes it possible to resist the premature fatigue of the material [54] and enhance both FS and FM [55]. To improve the FS of the PMMA, an in vitro study by Gray et al. showed the importance of precisely locating the area requiring consolidation. The reinforcement entails the use of a glass fiber mesh of specific dimensions in order to effectively contribute to the increase in the FS of the prosthesis [56]. In the same way as improving the FS of PMMA, three copolymers were incorporated: butyl-methacrylate (BMA), ethyl-methacrylate (EMA), and isobutyl-methacrylate (IBMA), in different proportions (10%, 20%, 30%, and 40%). All the samples tested were found to be higher than those of the control group (PMMA without copolymer addition). The concentration of 40% was found to be significantly more effective with IBMA [57].

The increase in the FS and the IS of the groups tested would stem from the low participation of the acrylate groups during and after the polymerization [58]. Another study reported that the matrix of the chemical structure of PMMA remains unchanged, even when adding hydroxyethyl methacrylate (HEMA) and IBMA molecules to it, while improving the FS [59]. Another incorporation of 0.6% polyimide significantly improves the FS of the PMMA prosthesis by 13.5% compared to the control group. However, resistance to bending decreases beyond this dose [60].

Interesting research comparing the material of the polyamide-based prosthesis (Valplast International Corp., Northport, NY, USA) and the PMMA, both with the addition of E-glass fibers, nylon 6 or nylon 6.6, revealed several consistent improvements. The Valplast resin shows superiority in its level of resistance compared to PMMA with or without fibers, and the same result applies to its modulus of elasticity. The added fibers, however, improved the structural elasticity of the PMMA.

PEEK in a homogeneous form has limited mechanical properties. The rigidity of PEEK remains a limitation, and thus the risk of fracture persists in the presence of direct exposure to occlusal loads [61]. Also, to address this drawback, additions in the form of fibers or ceramic molecules are currently being tested. This is why researchers have sought to combine PEEK with other materials such as ceramic to improve its properties. Note, for example, the high-performance biopolymer (BioHPP^®^, Bredent medical GmbH & Co. KG Weißen-horner Straße 2, Senden, Germany), which is a PEEK-based polymer containing 20% ceramic fillers [40]. These particles have a size of approximately 0.3–0.5 μm and are evenly distributed in the PEEK matrix, which makes the material more resistant. The addition of 3% of Nano SiO_2_ to prepare a composite of SiO_2_/PEEK, based on a final mixture during melting, made it possible to obtain a good distribution of the nanoparticles. This mixture increased the transverse resistance of the composite and lessened the hydrophobicity of the material while reducing the surface roughness [62].

## 4. New Fabrication Processes Improve the Properties of Dentures

To improve the properties of dentures, for several years, an alternative to the conventional method using CAD/CAM technologies has been proposed [63,64].

The manufacture of complete dentures using the CAD/CAM process provides several advantages and fewer appointments with patients; moreover, these appointments are shortened, but there is also a possibility of digital archiving the different stages of the realization [65]. For digital RPDs, thanks to the CAD/CAM manufacturing methods, the artificial teeth are manufactured and adapted to the morphology of the residual natural teeth by imitating their shape and their size and by adapting the occlusal contacts. On the other hand, concerning the prosthetic base, this mode of manufacture from the PMMA discs provides physical and mechanical improvements and a better surface finish and antibacterial properties compared to head-cured PMMA [66]. Moreover, some authors report a high level of satisfaction among patients and dentists with CAD/CAM prostheses [67,68].

A recent meta-analysis argues for globally better mechanical properties of CAD/CAM PMMA resins in comparison with heat-cured PMMA resin. In this review, from 13 studies carried out only in vitro, the comparison between 222 samples of heat-polymerized PMMA and CAD/CAM resin blocks of PMMA gave the following results: There was no difference in FS between the two samples, while the FM and the surface roughness were better for the PMMA resin blocks [69].

### 4.1. Advantages for Milling Dentures

Patients, practitioners, and laboratory technicians unanimously perceive the advantages of CAD [67,68]. In many cases, removable prosthesis bases designed by CAD/CAM are milled in acrylic resin blocks. The latter, which are manufactured industrially, have the qualities (mechanical and physical) required for daily use. Thus, this material has low porosity and releases a small amount of the monomer. It is characterized by its retention, toughness, hardness, and resistance to bending [64,70]. Milling prostheses have superior mechanical characteristics (resistance to bending, resistance to fracture), color stability, and adaptation of the base compared with the impression prostheses (Table 3).

In the form of ready-to-use blocks, PMMA is industrially polymerized under ideal temperature and pressure conditions in order to limit deformation. This manufacturing method produces fewer residual monomers [84]. The denture resulting from the machining of PMMA blocks has a smooth surface, facilitating daily hygiene. Recently, in comparison with polymethyl methacrylate (PMMA), the fabrication of removable prostheses by CAD/CAM systems focused on four materials: fiber-reinforced composite (FRC), nano-zirconia (N-Zr), cobalt-chromium-molybdenum alloy (CCM), and PEEK. The thickness of the palatal zone was 1.0 mm for PMMA and PEEK and 0.5 mm for FRC, N-Zr, and CCM. Under a load of 200 N at the incisive papilla, the deformation of N-Zr and CCM was half that of PMMA. At this same level, no significant difference in deformation between PEEK, PMMA, and FRC was noted. Thus, whatever the material used, manufacturing by CAD/CAM makes it possible to reduce the deformation of the prostheses [85].

### 4.2. Indication for Printing Dentures

Prostheses printed using stereolithography (SLA) 3D printers have better resolution if the orientation of the print direction is tilted at 45° [85]. Concerning complete prostheses, the impression technique seems to be attractive, but it still requires progress concerning the materials and the methods used [6,86]. The fully digitized manufacture of RPDs is currently limited to Kennedy III/IV classes. For partially edentulous cases of Kennedy class I/II, the digital impression technique does not make it possible to register correctly according to the edges of the base and the displacement of the mucosa under the pressure of the prosthesis [87].

Printing by sintering or laser fusion (SLS) is faster than the other techniques but also more expensive. In dental prosthetics, 3D printing can produce a model (in wax or plastic) that can be transformed into a definitive prosthesis, or it can directly produce definitive parts in metal, resin, or ceramic. Currently, the extrusion technique, which is ideal for thermoplastic polymers, is mainly used with PEEK [88]. An in vitro comparison of the FS values of six resins for prosthesis bases made it possible to establish the following hierarchy: Machined resins (AvaDent and Polident) came out on top, followed by a conventional heat-cured molded resin (Vertex) and a 3D-printed resin (NextDent), while polyamide and another 3D-printed resin (Harz) had significantly lower FS values than conventional resins [89].

### 4.3. Manufacturing Specificity of PEEK

The 3D printing technique for PEEK produces the best result in terms of the desired resistance, both in bending and in tension. This superiority of 3D-printed PEEK over other techniques depends on parameters such as temperature and printing speed [90]. It is mainly in the field of removable partial dentures that PEEK provides advantages for the replacement of an RPD framework in combination with acrylic resin teeth and a basic prosthetic material. Indeed, thanks to its low specific weight, PEEK contributes to the lightness of the prosthesis. For implementation, PEEK is suitable for extrusion and injection molding processes, and it can also be used to manufacture turned or milled parts [91]. In addition, the fatigue resistance of BioHPP, which is very high (1200 N), seems to be satisfactory for many indications [92]. However, concerning RPD, the study showed that the in vitro retention strength and fatigue resistance of PEEK claps were inferior to those of metal claps. A total of 16 metal clasps (1 mm thick) and 32 PEEK clasps (1 mm or 1.5 mm) were subjected to an insertion/removal test on a metal crown for 15,000 cycles. The metal clasps had a significantly higher retention force than the PEEK clasps, regardless of size [93]. Another recent in vitro experiment using fatigue tests confirmed that PEEK resisted load values significantly lower than those of the Co-Cr alloy. However, these values remained compatible with the daily clinical use of an RPD [94], which could jeopardize the balance of Housset’s triad in the long term. The qualities sought for the hook are elasticity for the necessary retention arm but also stabilization and support for the reciprocity arm. Currently, these characteristics are better suited to metallic alloys (Co-Cr) than to PEEK. Since the study was conducted on metal crowns in vitro, further in vivo studies are needed to determine whether the strength of PEEK clasps is clinically sufficient or not. Both for milled and for pressed PEEK at a mold temperature of 200 °C, an improvement in tensile strength is observed [14]. According to these authors, materials such as PEEK with high flexibility are not ideal for a prosthetic base, but on the other hand, flexibility is essential for the clasps of RPDs. This finding agrees with the results of Ucar et al. [16]; thus, the bending modulus and rigidity are more important than high flexibility [16]. The PEEK polymer can be considered a more resistant material at the level of bypassing the labial and lingual frenulum than PMMA. PEEK has a higher Young’s modulus but lower flexural deformation than PMMA. Its deflection curve is weaker than that of PMMA. Thus, the effect of these two parameters can relieve the supporting tissues under the prosthesis of the functional load [14,95].

The manufacture of removable prostheses from PEEK through the modeling of mol-ten deposits is one of the additive methods that has already proven its worth. Indeed, the adaptation of the PEEK frame of removable prostheses is satisfactory. However, this result still needs to be confirmed in the long term [96,97].

## 5. Polymer Choice According to Indications

### 5.1. Chewing Efficiency

When masticatory efficiency is sought, PMMA remains the material of choice for obtaining the best result [98,99,100,101]. However, for some authors, the polyamide offers better stability and retention in a removable complete prosthesis [102,103,104]. Thermosetting acrylic resins, thanks to their moduli of elasticity, are more resistant to deformation compared to less rigid polyamides [105,106]. Other authors recommend increasing the thickness of the polyamide bases to obtain sufficient rigidity [107]. However, polyamide, because of its low flexural strength compared to PMMA, deforms during mastication [16]. In addition, the elasticity of polyamide, such as Valplast, leads to the mobilization of the prosthetic base during chewing [101]. In the presence of extreme biting forces, the flexibility of the polyamide bases explains the absence of fracture [108]. On the other hand, in adult humans, the biting force with full dentition is between 60 N and 305 N [109].

In the studies by Rismanchian M et al. [110] and Nick Polychronakis et al. [111], the average value for a complete Valplast prosthesis is about 220 N after hydro-thermocycling at 3000 cycles. These authors note a permanent deformation in the presence of extreme loads. However, the values recorded for the tested materials exceed the minimum accepted force values (55 N) proposed by ISO 1567. Concerning RPD, two thermoplastic materials, acetal (AC) and polyamide (PA), can compete with resins based on PMMA [101]. Anna Macura-Karbownik compared the chewing efficiency and occlusal forces in wearers of PMMA, polyamide, and acetal RPD [101]. The replacement of missing teeth with PMMA or acetal prostheses proved to be beneficial in terms of the masticatory efficiency and the occlusal force developed. However, no significant correlation was found between chewing efficiency and occlusal forces. Another finding from this study shed light on the performance of removable prostheses fitted with clasps made of materials with a low modulus of elasticity. Indeed, the latter are associated with chewing efficiency and weaker occlusal forces.

An in vivo study focused on cases of Kennedy Applegate class I in the mandible or maxilla. A comparative study between Co-Cr, PMMA, and Valplast made it possible to test the effectiveness of mastication. After having restored the posterior occlusion, the Co-Cr RPDs proved to be the most effective in restoring the function of mastication. For these authors, among the three materials tested, Co-Cr offered the best performance for older and frail patients concerning diet, thus allowing the body mass index to be maintained [112]. In removable partial prostheses, the low elastic modulus of PEEK offers a damping effect with respect to occlusal forces. For this reason and the lightness, these removable prostheses based on PEEK have been tested to overcome the disadvantages of metallic materials [12]. With zirconia-reinforced PEEK, hybrid resin is offered as an alternative to PMMA in patients developing significant occlusal forces or for removable prostheses that have already suffered multiple fractures [113].

### 5.2. Fracture Cracks in the Denture Base

Different conditions can be at the origin of the fracture of a removable prosthesis, including maladjusted occlusions, masticatory muscles developing powerful forces, the instability of the prosthesis, or poor adaptation on the support surfaces. In addition, Beyli and Von Fraunhofer et al. (1981) [114] mentioned patient stress, greatly diminished edentulous ridges, as well as fragility due to the design of the prosthetic base. Each of these conditions justifies the implementation of a new prosthesis with a reinforced material [114,115]. In vitro, after simulating fractures in samples (50 mm × 25 mm × 3 mm) of acrylic resin (PMMA), the repair made it possible to test three types of resin: thermo-, auto-, and photopolymerizable. This experiment made it possible to highlight the superiority of thermopolymerized resins that have a significantly higher breaking load (FS: 6.55 MPa under an 87.36 N load) compared to self-curing (4.72 MPa under 72.94 N) and light-curing resins (4.06 MPa under 57.51 N) [116]. A review of the literature confirmed that the use of thermosetting PMMA implemented by compression using a water bath is very widespread [4,117]. Although for more precision, in vitro studies showed that PMMA has a high modulus of elasticity (0.85 ± 0.27 GPa for injection-molded PMMA base material SR-Ivocap) compared with conventional compression-molded PMMA (0.70 ± 0.23 GPa; Meliodent) [34,82].

However, in the presence of a fracture of the PMMA base, repair using a fiberglass mesh has given rise to several in vitro studies, testing the resistance as a function of the applied load. Flexural strength tests were carried out in vitro on 150 samples of heat-cured acrylic resin. The fiber mesh in the tension area of the PMMA specimens improved the flexural strength of the repair. However, the mesh and the dimension of the mesh integrated in the resin are essential to obtain a resistance to bending of the fractured prosthesis. Specimens repaired with the 20 mm fiber mesh placed in the tension zone showed the highest average FS with thermal cycling/non-thermal cycling [56,118]. The PEEK polymer could be considered as a resistant material to notch concentrations, as it revealed a higher Izod impact strength than the PMMA [96].

Recently, an in vitro study tested different bonds between the resins for the base of the removable prosthesis with, on the one hand, the prefabricated teeth (acrylic, composite, nanohybrid, and reticulated) and, on the other hand, the teeth produced by CAD/CAM. Bonding with a cold, hardened resin should be avoided when attaching prefabricated teeth to a denture base. Indeed, cold-cured base resins are not able to diffuse effectively into the prosthesis from the surface of the tooth. Regarding CAD/CAM (milled) and thermoset denture base resins bonded to different types of prefabricated teeth, they exhibit similar shear strength values [70].

## 6. Future Prospects

Continual progress is being made in the incorporation of nanoparticles into the polymer for therapeutic purposes. These modified materials have ushered in a new field of investigation that can improve both the prevention and treatment of stomatitis [119,120]. Another future challenge may be represented by 3D printing technologies and innovative 4D printing strategies. Thus, under the label of “intelligent materials”, it is envisaged that an inert object will be able to modify the behavior of its 3D shape over time. Four-dimensional printing uses stereolithographic principles. The influence of UV light is applied layer by layer to act on the hardening of the material. This is because the thermomechanical characteristics of memory polymers, called “intelligent material”, differ from those of ordinary 3D printing materials due to the change in shape [121]. Beyond 3D printing, 4D printing incorporates the additional dimension of time. This parameter reflects the ability of the material to deform over time. Thus, its influence within the oral cavity can be measured through several fluctuating factors such as pressure, air, heat, and saliva. The goal is to take these different parameters into account during the manufacturing process so as to improve the desired performance [122].

The 4D printing applied to the removable prosthesis makes it possible to adapt to the teeth and the mucous membranes but also to the constraints in the oral cavity. Four-dimensional printing uses smart materials for prosthetics adapting to the forces of bite, age, and diet. Shape memory polymers from 4D printing offer hope for improvements by increasing stiffness and having a faster reaction speed.

## 7. Conclusions

The ideal biomaterial combining the excellent characteristics of resistance, elasticity, and tolerance with oral ecology still requires improvements and in vivo applications. The choice of an appropriate polymer (PMMA, polyamide, or PEEK) for the base of a denture depends, first of all, on the mechanical properties sought. Concerning the manufacture of dental prostheses in the laboratory by the impression technique, several types of performance in the mouth concerning printed PMMA remain inferior, such as resistance to bending and fracture, compared to machined prostheses.

Removable dentures made of polyamide offer satisfactory results in terms of comfort and esthetics. But concerning their chewing efficiency and the degree of microbial colonization, improvements are necessary before they can compete with PMMA prostheses. They also remain confined to small recessed gaps or are used in combination with a metal frame to compensate for distal gaps. In the presence of a total loss of teeth, the mechanical properties of the polyamide, concerning mastication, limit the performance.

PEEK seems to be an interesting alternative to the use of alloys (Cr-Co) for removable partial prostheses with reinforcement. However, improvements are needed before they can compete with metal alloys. Thus, it is desirable to increase the thickness of the PEEK prosthetic base.

The second part of this review deals more specifically with the interactions between the oral environment (tissue, saliva, microbiota, pH) and the surface state of the different polymers—factors that will help us finalize our choice of polymer. Certain general pathologies affecting the edentulous patient can also influence this choice. Thus, for the treatment of denture stomatitis (DS), the preventive or curative therapeutic indications of these different materials remain to be defined in vivo.

## Figures and Tables

**Table 1:** Comparison of mechanical properties of PMMA, polyamide, and PEEK in in vitro studies.

Polymeric Biomaterials	Various Mechanical Properties							
	Tensile Strength (MPa) 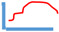	Elastic Modulus (GPa, ISO 2 GPa) Young’s Modulus 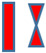	Flexural Strength (MPa) ISO More Than 68 MPa 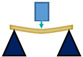	Compressive Strength MPa 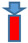	Elongation at Break (%) 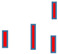	Flexural Modulus (1.2–2.2 GPa) 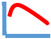	Impact Strength (KJ/m^2^) 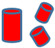	Hardness (kg/cm^2^ or VHN) 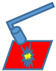
Heat-cured PMMA Meliodent: compressive; SR ivocap: injection molded; Lucitone 199	Mushin et al. [14]: PMMA HC (65 ± 5) PMMA pressed: (68 ± 9)	Mushin et al. [14]: PMMA HC (3.63 ± 0.02) PMMA pressed (3.78 ± 0.02) Zafar MS et al. [15]: HC PMMA (3.89 ± 1.32)	Ucar et al. [16]: Meliodent (81.1 ± 1) Ucar et al. [16]: SR ivocap (69.8 ± 1.4) Machado et al. [17]: Lucitone 199 (87.12 ± 8.1) Shrivastava et al. [11]: Lucitone 199 (84.05)	Neshati et al. [18]: Meliodent (71.9 ± 5.3)	Alla et al. [19] (4)	Ucar et al. [16]: Meliodent compression (1.70 ± 0.23) Ucar et al. [16]: SR ivocap injection (0.85 ± 0.27)	Al-Dwairi [20] et al.: Meliodent (14.75)	Ucar et al. [16]: Meliodent compression (16.9 ± 1.0 kg/cm^2^) Ucar et al. [16]: Sr Ivocap injection (13.5 ± 1.4 kg/cm^2^)
Polyamide Valplast Lucitone FRS Flexite supreme Deflex, Breflex	Takabayashi et al. [21]: Valplast (45) Lucitone FRS (70) Flexite supreme (75)	Takabayashi et al. [21]: Valplast: (0.82 ± 0.11). Lucitone FRS: 1.63 ± 0.08). Flexite supreme (1.57 ± 0.11) Soygun et al. [8]: Valplast values were lower than those of PMMA conventionnel	Ucar et al. [16]: Deflex (78.3 ± 1.0) Yunus et al. [22]: Lucitone FRS is significantly lower than Meliodent and comparable with Lucitone 199. Takabayashi et al. [21]: Valplast, Lucitone FRS, and Flexite Supreme were lower according to the ISO standard (higher flexibility).	Abhay et al. [23]: Valplast (NR) Wadachi et al. [24]: Valplast (NR)	(11.94 ± 0.14)	Ucar et al. [16]: Deflex injection (0.70 ± 0.13) Yunus et al. [22]: Lucitone FRS (1.71)	Soygun et al. [8]: Valplast was higher than PMMA conventionnel.	Ucar et al. [16]: Deflex injection (7.5 ± 1.0 kg/cm^2^)
PEEK Carbon-reinforced (CFR-PEEK) Maloo et al. [25] 2022	Mushin et al. [14]: PEEK milled (Invi-bio and JuvoraLtd UK) (118 ± 5), PEEK pressed (97 ± 4) Maloo et al. [25]: PEEK (100, 69)	Mushin et al. [14]: PEEK milled (5.59 ± 0.03), PEEK pressed (4.93 ± 0.02) Maloo et al. [25] PEEK (3, 5)	Shrivastava et al. [11]: PEEK (183.3 ± 4.79) Maloo et al. [25]: PEEK. (163, 88).	PEEK, ISO 604 [26] (120) Maloo et al. [25] PEEK. (118–169)	PEEK, DIN ISO 527 [27] (20)	PEEK, DIN ISO 527 (3.7)	Mushin et al. [14]: PEEK milled (4 ± 0.1), PEEK pressed (4.8 ± 0.4)	Maloo et al. [25]: PEEK (26–29 VHN)

PMMA, poly-methyl-methacrylate; PEKK, polyether ether ketone; ISO, international organization for standardization; DIN, deutsches institut für normung; HC, heat-cured; NR, not reported; VHN, vickers hardness number; TS, tensile strength; FS, flexural strength; CS, compressive strength; EB, elongation break; FM, flexural modulus; EM, elastic modulus; IS, impact strength; H, hardness. The superiority of the physical characteristics of PMMA (TS, EM, FS, FM, H) compared to those of polyamides justifies its indication for a prosthetic base for long-term use. The properties of polyamides (EB, IS) provide flexibility and explain their use for temporary removable prostheses. PEEK with a low impact strength is indicated mainly for the frameworks of removable partial prostheses thanks to its properties (TS, EM, FS, CS, EB, FM).

**Table 2:** In vitro clinical properties (effect of aging) of PMMA, polyamide, and PEEK.

Polymeric Biomaterials	Clinical Properties				
	Density (g/cm^3^) at Room Temperature	Water Absorption, ISO (<32 μg/mm^3^)	Solubility, ISO (<1.6 μg/mm^3^)	(%) and Time for Water Absorption at Saturation	Roughness (below Threshold of Accepted Norm of 0.2 μm)
PMMA heat polymerized	Mark et al. [41]: PMMA (1.18) Kutz et al. [42]	Nguyen et al. [43]: SR Ivocap HIP (25.8)	Nguyen et al. [43]: SR Ivocap HIP (<0.6) slight increase in weight (*p* < 0.5)	Nguyen et al. [43]: SR ivocap HIP 32 days Hamanaka et al. [44] PMMA 30 days	Al Dwairi et al. [45] 2019. Meliodent (0.22 ± 0.07) Sultana N et al. [46] 2023 SR Ivocap HI (0.0669 ± 0.02 µm)
Polyamide molded injection	Nguyen et al. [43]: polyamide (1.14)	Nguyen et al. [43]: Breflex (30.4), Valplast (13.6) Tagabayashi et al. [21]: Valplast (17). Lucitone FRS (39); Flexite supreme: (13).	Nguyen et al. [43]: Breflex and Valplast (net increase in weight) (*p* < 0.5) Shah J et al. [47] Flexite < PMMA Acron	Nguyen et al. [43]: Breflex 45 days Valplast 35 days (3.0) Lai YL et al. [48] Polyamides. 56 days	Abuzar et al. [49]: Flexiplast unpolished (1.11 ± 0.17), polished (0.14 ± 0.02); still noticeably rougher (>3 times) than the acrylic after polishing Sultana N et al. [46] 2023 Macro Flexi (0.1971 ± 0.02 µm)
PEEK Bio HPP (ceramic-reinforced), Finoframe 100% PEEK, Juvora medical 100% nature.	Maloo et al. [25]: PEEK (1.30–1.54) Skirbutis et al. [40]	Maloo et al. [25]: PEEK (0.1–0.5) Liebermann et al. [39]	Maloo et al. [25]: PEEK (<0.03)	Porojan et al. [50]: Bio HPP Finoframe PEEK Juvora medical PEEK 7 days (0.21–0.27); the weight changes in subsequent weeks were lower than 0.05%.	Porojan et al. [51]: Bio HPP (0.09 ± 0.01). Finoframe (0.08 ± 0.01). Juvora (0.08 ± 0.01)

PMMA, poly-methyl-methacrylate; PEKK, polyether ether ketone; ISO, international organization for standardization; %, percentage; HIP, high-impact polymer; HPP, high-performance polymer; FRS, super-flexible resin. According to ISO 1567 [52], the increase in the bulk density of dental polymers per unit of volume (water absorption) should not exceed 32 μg/mm^3^. According to ISO 1567, the acceptable solubility is 1.6 μg/mm^3^ for heat polymers. The three polymers (PMMA, polyamide, and PEEK) comply with ISO water absorption and solubility standards. The time for water absorption at saturation is better for PEEK than for PMMA and polyamide. The threshold roughness standard of 0.2 μm is accepted for PMMA and PEEK but not for polyamide.

**Table 3:** Physical comparison parameters between HC PMMA, 3D-printed resin, and milled PMMA in in vitro studies.

	Parameters					
Polymeric Biomaterials	Contact Angle (Zissis et al. [71] 2001) 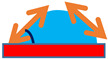	Surface Roughness (Ra) 0.2 µm Acceptable (Kul et al. [72] Dent 2016) 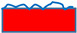	Vickers Hardness Number (VHN) 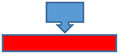	Flexural Strength MPa 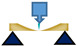 ISO: 65 MPa (Prpic et al. [70] 2020)	Flexural Modulus 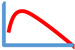	Impact Strength kJ/m^2^ 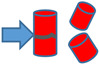
PMMA heat-polymerized Meliodent	Al Dwairi et al. [20] 2022: Meliodent (66.71 ± 3.38°)	Al Dwairi et al. [20] 2022: (0.22 ± 0.07 µm)	(18.11 ± 0.65) (differs with level of residual monomers) Al Dwairi et al. [20] 2022. Increased with copolymer Kiran et al. [73] 2021	Al Dwairi et al. [20] 2022: (92.44 ± 7.91)	Al Dwairi et al. [20] (2.084.99 ± 180.33 MPa)	Al Dwairi et al. [74] 2020): (16.64 ± 1.69 kJ/m^2^) (14.76 ± 2.11 kJ/m^2^)
3D-printed resin NextDent Dentona Asiga	Al Dwairi et al. [20] 2022: NextDent: (72.73 ± 2.10°), Dentona: (70.20 ± 2.43°) Asiga: (73.44 ± 2.74°)	Al Dwairi et al. [20]: Nextdent: (0.22 ± 0.07 µm) Dentona: (0.21 ± 0.06) Asiga: (0.19 ± 0.03 µm)	Al Dwairi et al. [20]: Dentona: (16.41 ± 0.96). Asiga: (16.24 ± 0.79) Next Dent: (16.20 ± 0.93)	Al Dwairi et al. [20]: Dentona: (81.33 ± 5.88). Asiga: (79.33 ± 6.07) Next Dent: (74.89 ± 8.44)	Al Dwairi et al. [20]: Nextdent > 2 GPa Dentona and Asiga < 2 GPa with bending before fracture. Dentona and Asiga exhibited considerable bending before fracture	Al Dwairi et al. [20]: Dentona: (17.98 ± 1.76 kJ/m^2^) Asiga: (16.76 ± 1.75 kJ/m^2^) Next Dent: (1.20 ± 0.69 kJ/m^2^)
Milled PMMA AvaDent Tizian-Shütz	Al-Dwairi et al. [45] 2019: Avadent: (72.87 ± 4.83°) Tizian-Shütz: (69.53 ± 3.87°)	Al Dwairi et al. [45] 2019: Avadent: (0.16 ± 0.03 µm) Tizian-Shütz: (0.12 ± 0.02 µm)	Al-Dwairi et al. [45] 2019: Avadent: (20.62 ± 0.33) Tizian-Shütz: (19.80 ± 1.08)	Abualsau et al., 2020. [75]: High pressure and high temperature improved the mechanical properties FS of 3D-printed < FS of milled PMMA	NR	Al Dwairi et al. [74] 2020: (24.56 ± 2.63 to 29.56 ± 6.94 kJ/m^2^). Superiority of milled PMMA/3D-printed and HC-PMMA. (Abualsau et al., 2020 [75])
Results	Modified surface wettability varies with chemical composition, topography, and salivary pellicule.	Not significant, but differs between different polishing techniques (*p* > 0.05).	Measure the resistance material (*p* < 0.05). Milled PMMA had higher values/heat-polymerized PMMA (Prpic 2020 [70]) (Ayman et al. [76] 2017)	Measure compressive, tensile, and shear stresses of materials *p* < 0.05	Higher flexural strength is advantageous for rigidity and stiffness (*p* < 0.05).	No statistically significant difference between Meliodent and 3D-printed resin (*p* < 0.05)
Effects	High hydrophobicity of 3D-printed denture base increases retain stain, plaque, and water sorption more than HC PMMA (Al-Dwairi et al. [45] 2019, Teixeira et al. [77] 2023, Meirowitz, A et al. [78] 2021)	Smooth denture surfaces reduce microbial adhesion and plaque (Choi et al. [79] 2020) (Foggi et al. [80] 2016)	Measure of how material resists plastic deformation during abrasion and mastication. Abdulwahhab et al. [81] 2013	Prpic et al. [70] 2020 found 3D (Next dent) had lower FS than milled PMMA, polyamide, and HC PMMA. Aguirre et al. [6]: rubber can favor resistance to deformation (Shaefer et al. [82]; 2010)	Ucar et al. [16] 2012: Not lower than 2 GPa	Reflects vulnerability of denture fracture. Superiority of milled pre-polymerized PMMA due to high temperature and pressure values (Prpic et al. [70] 2020). Improve IS by rubber particle; Rickman et al. [83] 2012.

PMMA, poly-methyl-methacrylate; HC, heat-cured; CA, contact angle; ISO, international organization for standardization; FM, flexural modulus, FS, flexural strength; NR, not reported. Superiority of PMMA HC polymerized for CA compared to 3D-printed resin and milled PMMA. No significant difference for RA between the three techniques. More significant values for VHN concerning milled PMMA. For FS and FM, the PMMA HC polymerized had better performance. The IS for the milled PMMA is better than that of 3D-printed or PMMA HC polymerized.

## Data Availability

Not applicable.

## Supplementary Materials

The following supporting information can be downloaded at: https://www.mdpi.com/article/10.3390/polym15173495/s1, Figure S1: Different polymerization techniques of PMMA, by compression or by injection molding. SR Ivocap (Ivoclar^®^), cooking in boiling water, a conventional compression molded heat polymerized (Meliodent^®^) (A); Pala X Press (Héraeus Kulzer^®^), vacuum injected (B); Acron M C I (GC Europe^®^) microwave, a compression molded microwave-polymerized (Acron MC^®^) (C); Swiss-Jet-Press (Condylator service^®^) injected pressed technique (D). Figure S2: Polymers materials, poly-methyl-methacrylate (C5O2H8)n (A,B); polyamide valplast CO (OH2)11NH)n (C,D); polyetheretherketone (-C6H4-O-C6H4-O-C6H4-Co-) (E,F).
