# Supplementary material for: Different Polymers for the Base of Removable Dentures? Part I: A Narrative Review of Mechanical and Physical Properties
Source: Polymers (Basel). 2023 Aug 22;15(17):3495. doi: 10.3390/polym15173495 (PMC10490543; doi:10.3390/polym15173495)
Supplement: Supplementary file 1 [file polymers-15-03495-s001.zip › polymers-2507978-supplementary.pdf]

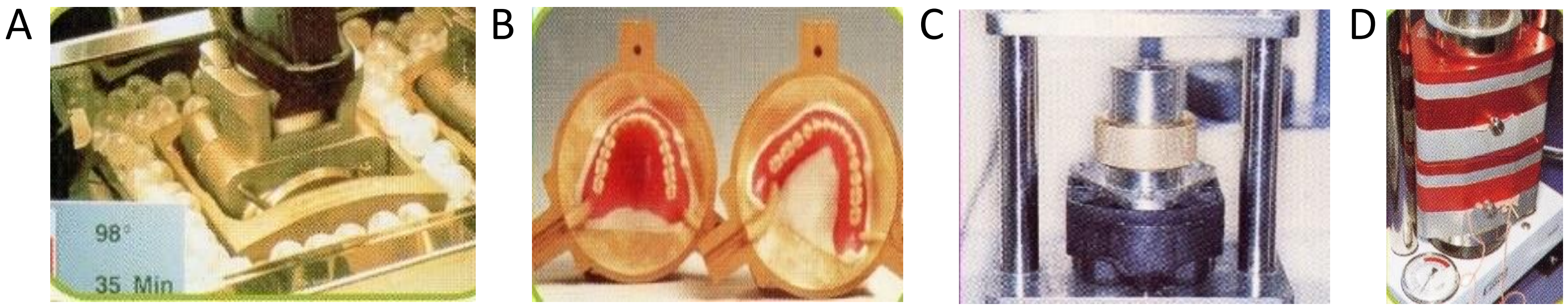

Figure S1: Different polymerization techniques of PMMA, by compression or by injection molding. SR Ivocap (Ivoclar®), cooking in boiling water, a conventional compression molded heat polymerized (Meliodent®) (A); Pala X Press (Héraeus Kulzer®), vacuum injected (B); Acron M C I (GC Europe®) microwave, a compression molded microwave-polymerized (Acron MC®) (C); Swiss-Jet-Press (Condylator service®) injected pressed technique (D).

A B C D E F

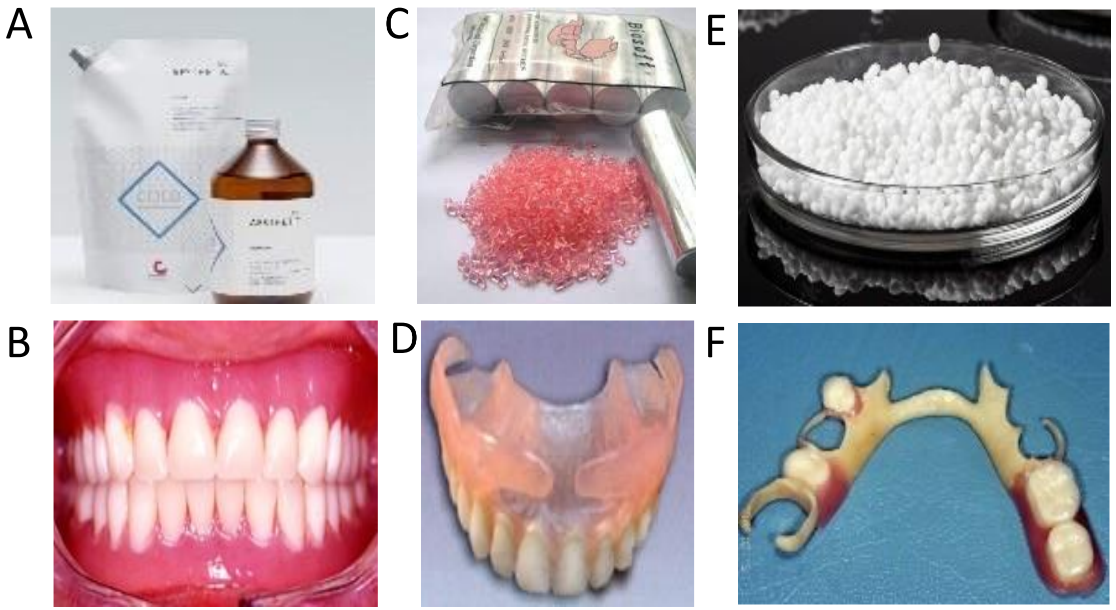

Figure S2: Polymers materials, poly-methyl-methacrylate (C5O2H8)n (A,B); polyamide valplast CO (OH2)11NH)n (C,D); polyetheretherketone (-C6H4-O-C6H4-O-C6H4-Co-) (E,F).
